# Transnational comparison of the impact of COVID-19 on medicolegal death investigations and the administration of justice: Early stages of the pandemic

**DOI:** 10.1177/00258024231182361

**Published:** 2023-06-20

**Authors:** Vienna C Lam, Steff King, Sheryl C Fabian, Gail S Anderson

**Affiliations:** 1 School of Criminology, 1763 Simon Fraser University, Burnaby, Canada; 2 Centre for Forensic Research, 1763 Simon Fraser University, Burnaby, Canada

**Keywords:** Coroner, medical examiner, forensic pathology, death investigation, COVID-19, pandemic

## Abstract

COVID-19 has had an unprecedented impact on arguably every sector of our criminal justice system. To assess the impact that this global health crisis has had on our medicolegal investigations and administration of justice during the early stages of the pandemic, this research aims to give voice to the lived experiences of medicolegal death investigators (coroners, medical examiners and pathologists). This research involved in-depth interviews and follow-ups with experienced personnel from Canada (3), Italy (1), the United Kingdom (1) and the United States (4). Results suggest that despite facing similar challenges, each individual office has had to develop their own strategies to overcome obstacles during the early stages of the pandemic. These results help identify overlapping areas for constructive policy and procedural changes, including recommendations for workflow adaptations, strategic partnerships and other approaches to best prepare for subsequent health crises.

## Introduction

During the onset of the COVID-19 pandemic in March 2020, medicolegal investigations were disrupted, roles redefined and the risk of contracting an aerosolized virus forced changes in how we manage our dead to protect practitioners. This mode of transmission and the highly infectious nature of SARS-CoV-2 rendered many aspects of mass fatality preparedness planning ineffective. Given that coroner and medical examiner systems investigate all unexpected and unexplained deaths, part of their work includes identifying the dead, collecting evidence from the deceased and serving as a conduit for the death investigation. This dynamic role involves working with police, hospital staff, body transport services, mortuary staff and the bereaved, among others making them a key conduit. The goal of this paper is to contextualize the challenges faced by coroners, medical examiners and pathologists from the United States and Canada during COVID-19 pandemic, including contributions from Italy and the United Kingdom. These results provide a snapshot of their lived experiences during the earliest stages of the pandemic, including what policies, procedures and strategic adaptations were implemented to meet new service demands.

## Literature

As of 4 May 2023, 6,921,614 people have died of COVID-19 – a number much higher than that for which many mass fatality programs have prepared.^1^ Mass fatality infrastructure requires a significant level of response preparedness and training for medicolegal professionals, amongst others. However, history has shown that instances of mass fatality – by natural disasters, events of terrorism or pandemics – often occur without warning. To contextualize this research, it is important to acknowledge reactions, responses and outcomes of exogenous shocks to coroners and medical examiners before COVID-19 began. These historical examples have informed modern response plans internationally and have exposed areas where modern coroners and medical examiners still need assistance.

### Historical mass fatality response

Disasters strike fast, requiring quick solutions from professionals, many without prior training or preparation. Historical mass fatalities have demonstrated that these reactive solutions place strain on coroner and medical examiner professionals as well as on their investigations of the deceased. For instance, overcapacity concerns demonstrated the need for creative solutions following the 1963 Indiana Coliseum explosion in the United States; morgues were quickly overrun, creating a need for storage outsourcing, including local hockey rinks.^2^ Many mass fatality incidents (MFIs) also resulted in the use of mass graves to either offset overcapacity or the spread of disease to living populations. For example, families had to sift through mass graves to locate loved ones in the aftermath of the 2010 Haiti earthquake,^3^ and many quick burials were unable to comply with religious practices in the aftermath of the 2014 Ebola outbreak from fear of exposure.^4^

Pressures for immediate response to mass fatalities can also diminish death investigative practices, such as identification and establishing the final manner and medical cause of death. Following the 11 September 2001 bombing of the US World Trade Center, the rush to collect DNA damaged the integrity of many samples, rendering them unusable for identification purposes.^5^ Further reports of the overwhelming pressure on hospital staff after the 2004 tsunami in India made post-mortem examinations impossible to complete, leaving many doctors and nurses responsible for assigning determinations as simply ‘death by calamity’.^5^

Existing literature also conveys the possibility of negative psychological and social impacts on medicolegal professionals who must respond to these mass fatalities. Brondolo and colleagues insist on workplace intervention programs after finding that continued work in mass fatalities may increase practioners’ risk for developing post-traumatic stress disorder and burnout.^6^ This is of particular concern when inexperienced response teams may be called upon in events of sudden disaster and overwhelming fatalities.^5,7^ To highlight the importance of examining these systems, existing literature suggests that mass fatality training and preparedness initiatives could minimize responder stress when incidents occur.^8,9^

In recent years, research exploring MFIs preparedness has uncovered consistent shortcomings in many coroner and medical examiner operations. Merril and colleagues reported that many medicolegal professionals perceived that their workplace was only ‘somewhat prepared’ to handle an event of a mass fatality.^10^ Additional concern arose when ‘less than half of the sample reported that their staff had been trained for MFI, and only about a quarter reported that staff had been trained for MFI with CBRNE [chemical, biological, radiological, nuclear, or explosive contaminants]’.^10^ Gershon and colleagues further reported that their sample of US coroner and medical examiner facilities often only had half of the operational capabilities (e.g., refrigerated storage, post-mortem examination facilities, transport) required for managing an MFI at any time.

### Responses to the coronavirus mass fatalities

On 27 January 2020, the Public Health Agency of Canada's Biosafety Advisory released its first publication of what was known about the coronavirus, which contained information about its human-to-human transmission modalities, symptoms and recommendations for diagnostic activities.^11^ Since then, they have released subsequent updates to include biosafety recommendations, development and implementation of standard operating procedures and policies related to all *in vitro* (i.e., culturing specimens, processing for distribution, etc.) and *in vivo* (preparing inoculum, inoculating animals and collecting specimens) activities with the virus. These documents serve as the groundwork for risk-mitigation strategies related to critical infrastructure in Canada.

The International Committee of the Red Cross published a call to action within a month of the pandemic being declared by the WHO which highlighted the importance of considering international humanitarian law when managing and protecting the dignity of the deceased.^12^ International human rights laws and international disaster response laws serve as measures of standards of care for the dead. Notably, these policies were made with the assumption that ‘in most cases, dead bodies do not spread diseases’.^12^ Given early reports of the potential of COVID-19 to still be transmissible from deceased premortem carriers and that the virus can be aerosolized during the natural process of body decomposition and putrefaction,^13^ what is clear is that even the most widely held international laws on management of the deceased need to be revaluated to fit the needs of a world engulfed in a pandemic. Once a person dies, they will no longer have any physiological functions, so methods of transmission from infected dead bodies should be further examined, as modality differs from that of the living.

This uncertainty in what legal provisions apply and mixed directives from authority figures has meant that medicolegal offices have had to make adaptations where possible and, in the case of the United States, have had to develop their own policies to mitigate risks of being overwhelmed and to protect the health and well-being of their staff. To augment what is known, this research aims to provide a real life and critical account of the early stages of the pandemic for medicolegal death investigators. Research questions include: *What impact has COVID-19 had on medicolegal death investigators? In what ways have Coroner/Medical Examiner Offices prepared for subsequent surges in deaths? What changes, if any, have been made to investigative mandates, provisions and/or policies?*

## Methods

The goal of this research was to document the lived experiences of medicolegal professionals and produce a series of recommendations for better medicolegal preparedness for future mass fatalities. To address the contextual differences between investigative systems, the sample includes professionals from four American states, two Canadian provinces, one English region and one Italian province, totalling responses from nine leading experts (we had two respondents from Ontario, Canada, that operated in different investigative jurisdictions).

### Recruitment strategy

Our recruitment strategy involved directly contacting personnel known to our research team that works directly within or in partnership with coroner and medical examiner offices, including forensic pathology units within hospital settings. We aimed to recruit a combination of coroners, medical examiners, pathologists and administrators (case managers) from around the world. This form of judgement (purposive) sampling technique was necessary to ensure that the study would involve a sample comprised of officials with access to privy knowledge, operational oversight and understanding of the broader implications that COVID-19 may have on criminal justice systems. As a non-probabilistic sampling method, we recognize that there may be inherent biases introduced, such as volunteer bias and group attribution error.^14^ This study does not aim to generalize findings, but instead, provide rich descriptions of the death management strategies taken amidst a world health crisis. Interviewees ranged from frontline workers to chiefs or directors. Some were affiliated with universities and held additional responsibilities, including instruction, as a clinician and/or conducted research. In a few instances, the interviewed participants were also part of mass disaster fatality response teams.

### Data collection and coding

One-hour semi-structured interviews were audio-recorded and transcribed, and participants were given the opportunity to supply supplementary information via email. NVivo 12.6 Plus (ed.) was used to strip identifying information from the transcriptions, coding themes and for the thematic analysis of guidance documents. The first set of interview questions asked about COVID-19 pandemic impact on their workplace and practices, including their access to equipment and resources, personal safety training and/or policies implemented. The second set of questions related to changes to death management practices, including challenges with storage capacities and their ability to determine the manner, medical cause and mode of death. Lastly, the third set of questions related to their emergent plans for the foreseeable future, what policies are being put in place to address future health and safety emergencies. Thematic analysis was selected to identify patterns and themes from the medicolegal professionals’ experiences.^15^ Inductive strategies allowed interview data to speak to the lived experiences of the respondents through their own voices.^16^ The coding process involved four rounds of data review, where common themes were identified across respondents, and follow-up interviews were employed to verify or clarify individual information. Select follow-up interviews were also held where discrepancies were discovered during the coding process. Four rounds of coding were necessary to reach feelings of saturation, in that no new information or further connections were being made.^17^ Regional and systemic differences were considered in the coding of these materials, including differences in health providers and the role of forensic pathologists in medicolegal investigations. The interviews and follow-ups took place in the months of June to August 2020, during the first wave of the COVID-19 pandemic, and all pertinent documentations were collected from May to September of the same year.

## Results

Coroners, medical examiners and forensic pathologists have had to quickly adapt to challenges caused by the pandemic and change their routine work to meet service demands. The following themes present the circumstances from which they have had to work during the early stages of the pandemic (March 2020–August 2020), as well as the strategies employed.

### Theme 1: COVID-19 impact on attending the scene

Under normal circumstances, coroners and medical examiners are often called to attend the scene to perform a preliminary assessment of the deceased body, as well as make an initial determination of whether this is a case that would fall under their mandate. Natural deaths related to old age or terminal illness which occur under the care of a physician (assuming there was no medical malpractice) are typically exempt. As COVID-19 cases increased, improving how this distinction was made was of utmost importance to limit unnecessary exposure. Death investigators in Arizona and Florida were still attending the scene, but all possible report writing and work that could be done from home was made remote. In Arizona, viewing scenes remotely meant that they were able to manage the sharp increase in workload. In response to asking whether they missed attending the scene, they responded:

Arizona participant: ‘I don’t like to go because there's less control; not of myself but of other individuals that might be at the scene. And then, of course, if you’re working on the body, you don’t know if they’ve been exposed… I try very hard to do it from my phone if I can…’

During the early stages of the pandemic, the medical examiner's office in Alabama had also switched to remote work when possible but continued to attend scenes. Masks and gloves were made available to death investigators. Although an updated guidance on the use of masks was released by the WHO on 12 June 2020,^18^ many jurisdictions had yet to adapt policies or put systems in place to enforce compliance. In Alabama, death investigators were told that using masks was at their discretion.

Alabama participant: ‘If they think it's appropriate, they’ll use it. If they think that it was going to antagonize the family or upset them, then they can choose to not use it. They can use their judgement and proceed. So far, no one in the office has been infected so it seems to be working’.

There was a real contextual difference between the communities being served, especially with regards to which cities have had ongoing anti-mask protests and which communities have had government officials openly declare anti-mask sentiments – all of which would make it more difficult to enforce compliance. Four participants stated that they believed that they were at greater risk of contracting COVID-19 during their everyday activities (such as going to purchase groceries), than when they were on the job attending scenes and/or working with the deceased in the mortuary.

Aside from difficulties working with families with anti-mask sentiments, additional personal protective equipment (PPE) has been reported to be unobtainable or uncomfortable. Global shortages and mandated public use of PPE resulted in increased costs. In warmer locations, the need for additional levels of PPE lengthened investigator's time spent in the field, but in the case of Florida, they reported that this delay has not damaged their ability to collect evidence.

Florida participant: ‘There is an added cost in both time and equipment. Additional PPE must be purchased and worn. At this time the appropriate PPE can be difficult to obtain. The additional PPE needed can be problematic due to environmental conditions. For example, today the humidity index is 105 degrees. It takes longer to process the scene because investigators are not able to remain in PPE for an extended time. Approximately 20 min in PPE under those conditions is the maximum, and a break is required to cool down. The reality is that it is slower and more costly process, but I do not feel like it has damaged our ability to collect’.

When it comes to risk-mitigation strategies, our participant from Ontario said that they benefited from taking a proactive approach by treating the pandemic as a mass fatality event from the early stages. This was evidenced by the early guidelines issued by the Office of the Chief Forensic Pathologist in March 2020.^19^

Ontario participant: ‘… there was a lot of preparation, both from a biosafety point of view with linking to the funeral section to remove the possibility of supplementary mortuary facilities, the death certification process was streamlined, so that accompanied by the fact that there was only a modest surge in actual mortality – the preplanning plus the… I think there were three things actually, there was the preplanning, there was good PPE procurement and management, and then there are good autopsy guidelines…

So those things that I’ve talked about, the policy environment, the PPE and the autopsy guidelines, those actually came out of some very discreet things. Number 1, very good communication between the coroners and the pathologists. Number 2, really good communication with the funeral sector, with the Bereavement Association of Ontario. Number 3, good communication with our public health partners, with coordinating efforts. And number 4, we are under the auspices of the Solicitor General and they have had an excellent Minister and ministerial level of response to this. So, it really created a really excellent environment for a public response’.

In the United Kingdom, remote scene viewings were seen as a necessity to offset the sudden surge of casework, particularly in London. In addition to the use of telehealth software that enabled coroners to make their assessment remotely with the help of care home staff members, the sudden surge of cases in London resulted in the development of Pandemic Multiagency Response Teams (PMART) to help alleviate the pressures related to attending potential crime scenes for initial determinations of deaths. Our UK respondent explained that PMART could ‘*immediately certify death because the numbers in London were significant. There were worries that [the] mortuary would be overwhelmed’.* Our UK respondent stated that being able to train paramedics who could attend the scene with police officers proved to be instrumental in maintaining workflow.

### Theme 2: COVID-19 Impact on Family Viewings and Identification of the Deceased

Generally, when a death occurs at home, identification of the deceased takes place through visual confirmation by other occupants or building managers. When the death takes place outside of the deceased's residence, the family may view the body at either the morgue or coroner/medical examiner suite. Not all hospitals are equipped with viewing rooms with appropriate glass barriers and pathways to limit the disruption to other hospital services. If there is no visual identification, then fingerprints and dental records are normally used to confirm tentative identification (e.g., driver's licence, name on prescriptions, etc.). Since COVID-19, visual identifications have been brought to a halt.

Among our nine participants, those from Alabama, Arizona and Ontario do not have viewing rooms. In British Columbia and Florida, families will see the dead in funeral homes, and that has not stopped, to their knowledge. In Italy and the UK, family viewings did happen in designated forensic suites prior to COVID-19. Since then, the UK has had to suspend all viewings and Italy, after suspending viewings for months, has just started allowing this practice once ‘*precautionary measures [were] adopted by all funeral workers and families (face masks, washing hands)’.* Only a limited number of relatives were allowed, and in many cases, family members would only be able to do so through a viewing room, without any physical contact with the deceased or funeral workers.

In Alabama, visual identification of the deceased still takes place in the form of photographs of either the person's face or tattoos. When asked about further testing for the unidentified and whether they have faced any delays, they reported that DNA analysis has always taken a couple of months to complete. Since COVID-19, Alabama has had success identifying three of their long-term cold cases through the National Missing and Unidentified Persons System (NamUs).

In addition to these aforementioned identification methods, places such as Arizona have a forensic artist in their employ who will create a rendition based on photographs of the deceased and have a forensic anthropologist construct a biological profile in the case of dismembered or highly decomposed remains. Since COVID-19, they report that ‘*unless it's muscle or blood, there's no one that can look at [DNA from dentition] for us so they go into the archive in hopes that someday we’ll be able to get it done’.* They stated that they don’t think the unidentified number has gone up (as of July 2020), but they ‘*expect that as this summer goes on, it will, and by the end of the year, we will probably have more [unidentified] than we usually do just because of the difficulty of outsourcing some of these [DNA] problems’.* When asked about why they believe this backlog for identifying the deceased has taken place, they stated that this has been an issue pre-COVID.

At the time of this interview, there was currently no research on how long the virus may still be transmissible from a deceased person in sequestered, enclosed conditions. As such, Florida made the decision that body bags containing unidentified deceased persons with potential SARS-CoV-2 infection should not be reopened. This limits the ability of investigators to make an identification, though it is unknown what number of cases has involved unidentified persons since the pandemic began.

Since March 2020, there have been reported increases in social isolation, which include self-imposed quarantines, job loss, school closures and other factors that lead to people self-isolating. As a result, one such effect of this alienation is delayed discovery of the deceased, whereby deceased bodies are found in a more advanced stage of decay than that pre-COVID. With decompositional change, valuable information is lost, and the typical scientific measures used to produce an estimate of when the person died, become increasingly unreliable.

United Kingdom participant: ‘More decomposed bodies at home. So, with decomposed bodies, visual identification is not possible. Dentistry is still a challenge. Fingerprints are not obtainable, so DNA has to be tested when someone has been in there a long time before we’re alerted to the death’.

### Theme 3: COVID-19 Impact on Determining the Manner and Medical Cause of Death

During the earliest stages of the pandemic, there were a lot of concerns related to how COVID-19 bodies should be safely handled. At the time of the interviews, the Centre for Disease Control (CDC) Guidance Postmortem Specimens Protocol had just been updated (15 June 2020).^20^ When pathologists realized that the use of a bandsaw could inadvertently aerosolize the virus, the process by which internal autopsies were performed had to be revised. In Italy, they were grossly overwhelmed, but have since built up their capacity to ensure that when an autopsy is necessary for the medicolegal investigation, they are able to do so in appropriate forensic suites.

New York participant: ‘Many COVID-19 positive non-homicide [autopsy] cases have not been performed, especially in offices that didn't have specific requirements to do autopsies in a safe environment (for example, negative pressure rooms). In those cases, a complete medicolegal death investigation, which generally includes a full postmortem examination, was not possible’.

United Kingdom participant: ‘There are some coroners, for example, with a hanging case, [they] may just do an external examination to match the ligature marks and maybe toxicology instead of embarking on a fuller examination. So you would target what you wanted out of the pathology exercise rather than doing a complete comprehensive examination – Unless it was forensic of course, we would only do it if it were a homicide suspicious and that would be done by our office by a registered forensic pathologist and we only have about 35 of those in the country’.

Italy participant: ‘The Italian Society of Legal Medicine promptly informed the Italian Ministers of Health and justice about the reduced ability to provide death investigations all over the country since most of the autopsy rooms in the Italian territory did not meet the [CDC] requirements for BSL3… One of the potentially negative impacts of COVID-19 to death investigation was certainly related in the beginning to the reduced ability to complete death investigations and to the reduced number of autopsies performed’.

As of summer 2020, the Italian participant reported that there were no issues with the collection of post-mortem and clinical and pathological specimens, and they were collected ‘*according to proper biosafety and infection control practices’.* Our Toronto participant reported that COVID-19 has not impacted their processing of homicide or criminally suspicious deaths, and that new safety measures were quickly adapted with the initial release of ‘*Guidelines for Medicolegal Autopsies in Ontario during the COVID-19 Outbreak’.*^19^ These guidelines included clear directives to suspend all autopsies unless there are extenuating circumstances (e.g., homicide cases), only conduct them in negative-pressured autopsy suites, use of N95 masks and PPE, using bone shears instead of electric oscillating saws, and to perform a nasopharyngeal coronavirus swab when the deceased is suspected of having COVID-19, among other measures. The rapid deployment of updated strategies and guidelines may have helped give Ontario health workers the time necessary to build up their capacity to process deaths in an expedient fashion so as to avoid being overwhelmed.

When asked about whether medical examiner and coronial systems are correctly classifying COVID-19-related deaths, the Ontario respondent said that:

Ontario participant: ‘We’re going to have to look at excess deaths on a daily basis or going to have to look over the period how many excess deaths we’ve had. And then see whether those are therefore COVID-related or whether they are related to COVID plus failing to get medical attention for diseases that they could have treated’.

This information has not yet been released to the public, so a closer look at death trends will have to be examined in the future to quantify death classifications:

United Kingdom participant: ‘And certainly we have had a couple of homicide inquiries… They are interested in the cause of death [in] any forensic recovery that may identify the perpetrator if the perpetrator has not already been identified or suspected’.

These post-mortem nasopharyngeal swab tests for coronavirus should not be used to determine whether the medical cause of death is coronavirus, as it can only speak to the presence of coronavirus in the body at the time of the swab. Also, as our UK participant points out, ‘*the fact that they might’ve had a test prove negative doesn’t mean that they couldn’t have caught it and have subsequently died from it’.* The same concerns over how these tests are being used in investigative procedures is echoed by our Italian participant:

Italian participant: ‘Therefore, a lot of clinical information were available including radiology. Worth mentioning is the fact that several deaths (actually under review) showed clinical and radiological findings consistent with severe pneumonia probably due to COVID-19 but swabs were negative for COVID-19’.

### Theme 4: COVID-19 Impact on Jury Inquests and Issuing Death Certificates

Although some courts have had to suspend all hearings, smaller, discrete inquests can still happen swiftly despite delays due to courts trying to adapt and implement safety measures. Places such as British Columbia have their own dedicated facilities to hold inquests. The UK participant reported still having around five hearings a day and that this was a new normal up until the end of August 2020; there, families are entitled to attend hearings, but there was a visibly lower attendance that summer. Attendees are required to practice good hygiene, use sanitizers and both gloves and masks are available, though this is not enforced. Remote hearings are also now possible. Doctors in the UK are able to certify deaths as natural deaths (e.g., deaths attributed to contracting SARS-CoV-2) so long as they have seen the patient within 14 days of the death, but with the enactment of the UK COVID Virus Act,^21^ this time limit has been extended to 28 days.

Canadian COVID-19 deaths typically occur under the care of a doctor thus the attending physician can sign off on the death, which appears to be a widely held practice, save for deaths under suspicious circumstances. In Arizona, our participant reported that they are not accepting jurisdiction in known COVID-19 cases. They believed that if they had made a different decision at the beginning of the pandemic, they would have been overwhelmed like jurisdictions that chose otherwise. They stated that DC has taken all cases to maintain consistency in death certificates, but their colleagues have expressed concerns about that decision. Respondents from Alabama, British Columbia, Ontario, Florida and the United Kingdom all discussed how digitizing the issuance of death certificates has helped them maintain consistency and also expedite the process – many expressed that they had made the transition to digitization prior to the coronavirus outbreak so the pandemic only served to accelerate that workflow change.

There are 25 medical examiner districts in Florida. The Florida Emergency Mortuary Operations Response System (FEMORS), situated at the University of Florida, houses several mass disaster teams and their mandate is to support the medical examiner systems, as well as hospitals and funeral homes. They will offer their ‘*assistance, whether they are medical examiner issues or not as long as there is interest to the state of Florida’.* FEMORS is active on a daily basis, which includes supplying personnel, equipment (e.g., sourcing a digital x-ray when the one at a medical examiner's office malfunctions) and since COVID-19, has also taken the lead in establishing new supply chains to provide necessary body bags and PPE to offices across the state. FEMORS is contracted to respond to 500 simultaneous deaths at any moment.

Due to Florida's vulnerability to natural disasters, Florida's mass disaster plan first came into effect in 1941, with the enaction of the Florida Civil Defense Council Act's Chapter 202.1.2 Florida Statutes.^22^ Since then, several statutes came to be, to create systems of support for mass disasters and other state-wide emergencies. Part of that system includes diverting cases once the manner of death has been determined if they fall outside the medical examiner system's mandate, as well as allowing doctors in hospitals to sign death certificates. During the early stages of the coronavirus pandemic, it was clear that the ‘*Florida medical examiner system isn’t really robust enough to handle 80,000 deaths in two weeks… Thankfully, the private Funeral Home industry in Florida is really robust’.* If the deceased was a known COVID-19 death, the body never enters the medical examiner system. Instead, the medical examiner office will certify the death based on the hospital doctor's reports.

## Discussion and recommendations

This pandemic has differed from other mass fatalities because our world is more populated and physically interconnected. Global travel results in increased risk, and as such, medicolegal systems must be able to deploy strategies quickly and efficiently, while taking the time to reflect on lessons of the past. There are many tangible social and legal ramifications that are predicated on how well these offices are able to navigate health crises. In this section, we will further discuss tales of caution and recommendations for mass fatality preparedness.

### Overcapacity concerns

The overwhelming number of cases demanded additional training of first responders to make death determinations at the scene and for doctors to sign death certificates in hospitals. This resembles the outsourced death determination duties given to hospital staff in the 2004 tsunami in India where doctors and nurses assigned cursory manners of death to offload cases.^5^ Given the already limited number of trained medicolegal professionals available, this all-hands-on-deck mentality works as a quick solution to process cases quickly. However, existing literature posits that inexperienced responders, particularly those who have not worked with the deceased before, could suffer additional stress and psychological trauma.^5–7^

Mass fatalities also place additional strain on already limited resources such as PPE and storage facilities for the deceased. The Florida participant indicated that not only was PPE expensive but it was also difficult to obtain. They also had to take on the role of securing what they need for their own operations, as well as support other smaller offices. With demand high across the world, limited resources have the potential to both lengthen death investigations and place medicolegal professionals at risk of exposure if they must work without it. Recalling Gershon and colleagues’ findings that US coroners and medical examiners reported limited access to resources and facilities generally, it is concerning to think that many medicolegal facilities likely started the pandemic with already insufficient resources.^23^

Despite the difficulties identified with overcapacity, there does appear to have been lessons learned from the past. Some participants indicated that existing preparedness plans helped them build their capacity to levels where they could continue modified medicolegal investigations, particularly for homicide cases. There was also evidence of practitioner ability to assess the risk of COVID-19 exposure from the deceased and respond accordingly. Of note, the Arizona participant acknowledged the loss of information that comes from reactionary mass burying of the dead during fears of outbreak – this is a significant change of mindset from past mass fatalities given that mass burials became the solution to decrease the risk of exposure to disease.^4^ Others also figured out how to allow families to identify the deceased through well-defined precautionary measures or virtual technology. These instances show that in adversity, there are still solutions.

### Investigative methods

Identification problems are also a continued problem, as they were in past mass fatalities. Participants acknowledged that there were notable changes to identification procedures and decreased abilities to conduct post-mortem examinations. Outsourcing DNA analyses have slowed with laboratory shutdowns during the pandemic indicating to some medicolegal professionals that this may lead to an increase in unidentified cases over time. This was also a concern following the 11 September US World Trade Center terrorist attack in 2001, where limited DNA technology led to a still high number of unidentified cases.^24^ It is the hope that with the advanced forensic technology of today, and with laboratories reopening, current cases will not remain unidentified for so long.

The concern for exposure via aerosolization has also restricted post-mortem examinations for cases that do not immediately appear as homicide – a manner of death determination that may rely on virtual communication or inexperienced responders at the scene. The decreased information that comes from these measures may impact both current understanding of mortality statistics and criminal justice proceedings that require such evidence. There has been emergent research indicating COVID-19's impact on the criminal justice system across the world already,^25–27^ indicating that changes in how we gather information impacts how proceedings occur. As death investigation is a crucial part of the criminal justice system, we must consider how these investigative changes may also impact case interpretations and future understandings of crime projections during COVID-19.

Despite these concerns, there is still value in the quick development and refinement of existing investigative practices during COVID-19. Virtual communication during death investigations created the ability for coroners to view the scene more frequently in real time. This technology has also allowed a wider audience to attend public inquests through videoconferencing which may increase public awareness of investigative proceedings. Only time can tell if these changes to death investigation practices will impact criminal justice tidings, an avenue that future research will hopefully examine, but it remains clear that there are still many changes needed to assist medicolegal professionals during COVID-19 and beyond.

### Recommendations

Mass fatalities have happened before COVID-19 and will happen again, meaning what we have learned from the past and the present will undoubtedly help us in the future. While we cannot predict the nature or size of the next event, and although our sample size is quite small, the themes suggested in this research have indicated both instances of success and frustration from medicolegal professionals which may benefit others in a similar situation. Based on the lessons learned from our participants, we suggest six key recommendations for the future:

1. Increase COVID-19 testing capacity by partnering with local universities. Many universities have pathology departments. The most hard-hit locations quickly adapted to a policy where they test all incoming deceased persons and are able to receive results within 24 h. This is particularly important in cases where rapid tests have yet to be developed.
2. Train paramedics to conduct initial assessments. When London was becoming quickly overwhelmed by the surge in cases during the early stages of the pandemic, their decision to train and create PMART proved extremely useful because they were able to divert cases away from their medicolegal system. This process also allowed for telehealth determinations of death when the paramedics needed medicolegal support.
3. Adapting parcel pick-up machines for personal affects. When personal belongings of the deceased need to be returned to the next-of-kin, consider implementing parcel pick-up lockers outside a police station or mortuary home, which limit physical contact with the bereaved.
4. Suspend or divert family viewings to mortuary homes. Virtual viewings can be facilitated. Bereavement services should be updated to discuss safer alternatives.
5. CDC Biosafety Lab 3 measures are a must. Although the guidelines released are recommended best practices, our Italian participant's account of their devastation speaks to the importance of only conducting autopsies in forensic suites with negative air pressure and other risk-mitigating features.
6. Cohort scheduling. Many offices adapted cohort scheduling models, similar to those of police agencies where workers are assigned to small task teams and work on a rotating schedule to limit contact between teams.

## Conclusion

There are real concerns about the potential for miscarriages of justice because of diminished investigative due diligence. Many communities around the world have yet to face the overwhelming burden of COVID-19 on their criminal justice and public health sector, so these recommendations may prove useful in anticipation of future pandemics. The aim of this work was to capture the challenges faced during the early stages of the pandemic, offer policy recommendations based on lessons learned by coroners, medical examiners and forensic pathologists and offer credible, authentic accounts of experiences, directly from the voices from this community of frontline workers. We end with a quote by our participant from Arizona, ‘*[COVID-19] is a great equalizer and it has demonstrated where the gaps are and allowed us the opportunity to address those gaps’.* It is this optimistic pragmatism that is going to help us this global health crisis.
